# Supplementary material for: An examination of school reopening strategies during the SARS-CoV-2 pandemic
Source: medRxiv. 2021 Mar 19:2020.08.05.20169086. Originally published 2020 Aug 6. Preprint. [Version 2] doi: 10.1101/2020.08.05.20169086 (PMC7418733; doi:10.1101/2020.08.05.20169086)
Supplement: Supplement 1 [file media-1.pdf]

Supporting Information

Compartmental Model

The ordinary differential equations (ODEs) describing cohort k are given by

$$\begin{array}{ll} \text{Children} & \text{Adults} \\ \dfrac{\mathrm{d}S_{1k}}{\mathrm{d}t} = -\lambda_{1k} S_{1k} & \dfrac{\mathrm{d}S_{2k}}{\mathrm{d}t} = -\lambda_{2k} S_{2k} \\ \dfrac{\mathrm{d}E_{1k}}{\mathrm{d}t} = \lambda_{1k} S_{1k} - \sigma_1 E_{1k} & \dfrac{\mathrm{d}E_{2k}}{\mathrm{d}t} = \lambda_{2k} S_{2k} - \sigma_2 E_{2k} \\ \dfrac{\mathrm{d}I_{1k}}{\mathrm{d}t} = \sigma_1 E_{1k} - \gamma_1 I_{1k} & \dfrac{\mathrm{d}I_{2k}}{\mathrm{d}t} = \sigma_2 I_{2k} - \gamma_2 I_{2k} \\ \dfrac{\mathrm{d}R_{1k}}{\mathrm{d}t} = \gamma_1 I_{1k} & \dfrac{\mathrm{d}R_{2k}}{\mathrm{d}t} = \gamma_2 I_{2k}, \end{array}$$

where the left and right columns correspond to SEIR compartments for children and adults, respectively.

Basic reproductive number

We characterize the basic reproductive number $\mathcal{R}_0$ indicative of growth potential of an infectious disease. Specifically, $\mathcal{R}_0$ quantifies the expected number of secondary infections due to a single infected within a completely susceptible population. The threshold $\mathcal{R}_0$ value of 1 marks the boundary between explosive growth ($\mathcal{R}_0 > 1$) and decline of an epidemic to extinction ($\mathcal{R}_0 < 1$). We derive $\mathcal{R}_0$ for our stratified SEIR model using the next generation method outlined by Diekmann, Heesterbeek, and Roberts [1]. Near a disease-free equilibrium point, it is reasonable to linearize dynamics by taking the initial proportion of susceptibles, $S_{jk}(0)$, approximately equal to its maximal value defined by the population's demography, q_{jk}. Thus, the transmission and

transition operators $\boldsymbol{T}$ and $\boldsymbol{\Sigma}$ are given by the matrices

$$\boldsymbol{T} = \begin{bmatrix} 0 & \alpha_{11}\beta_{11}q_{11} & 0 & \alpha_{11}\beta_{12}q_{11} \\ 0 & & 0 & 0 \\ 0 & \alpha_{11}\beta_{21}q_{21} & 0 & \alpha_{11}\beta_{22}q_{21} \\ 0 & & 0 & 0 \end{bmatrix} \qquad \boldsymbol{\Sigma} = \begin{bmatrix} -\sigma_1 & 0 & 0 & 0 \\ \sigma_1 & -\gamma_1 & 0 & 0 \\ 0 & 0 & -\sigma_2 & 0 \\ 0 & 0 & \sigma_2 & -\gamma_2 \end{bmatrix},$$

based on the infectious subsystem defined by $\boldsymbol{x} = [E_{11}, I_{11}, E_{21}, I_{21}]^\top$ for a single cohort. Together, these linear operators recover the linearized subsystem $\frac{\mathrm{d}\boldsymbol{x}}{\mathrm{d}t} = (\boldsymbol{T} + \boldsymbol{\Sigma})\boldsymbol{x}$. Following Diekmann et al. [1], $\mathcal{R}_0$ is taken as the spectral radius of the next generation matrix with large domain, $-\boldsymbol{T}\boldsymbol{\Sigma}^{-1}$. In the case of multiple cohorts, the structures of $\boldsymbol{T}$ and $\boldsymbol{\Sigma}$ as given are repeated in a tiled fashion, with the appropriate changes in indices for $\alpha_{k\ell}$ and q_{jk}.

Additional details on model assumptions

Unless specified otherwise, model parameters are taken as defined in Table 1 in the main text.

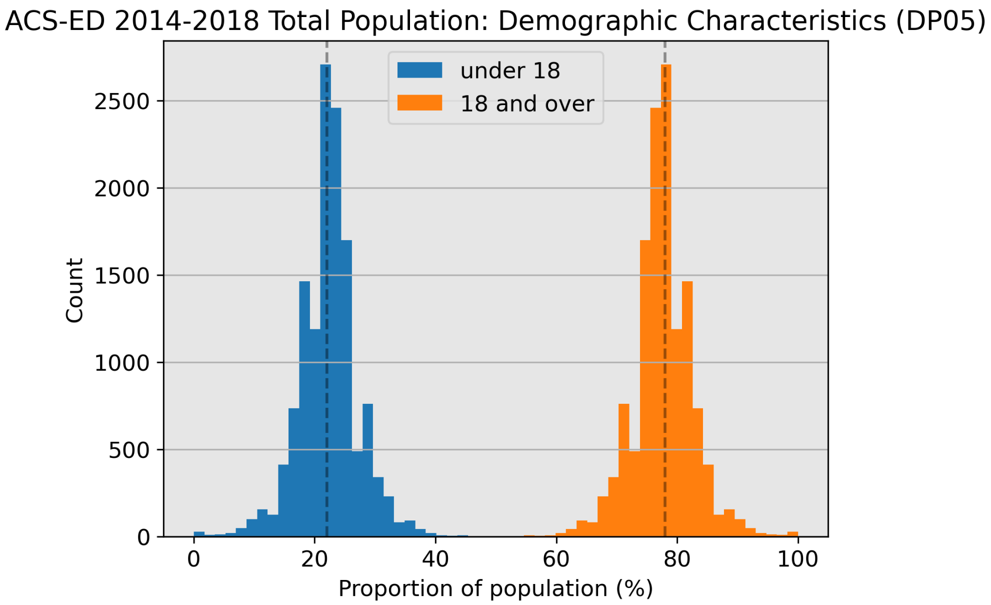

Fig S1. Population demography across school districts in the United States. Vertical lines denote the median proportion for both groups, 22% for individuals under 18 and 78% for individuals 18 and over.

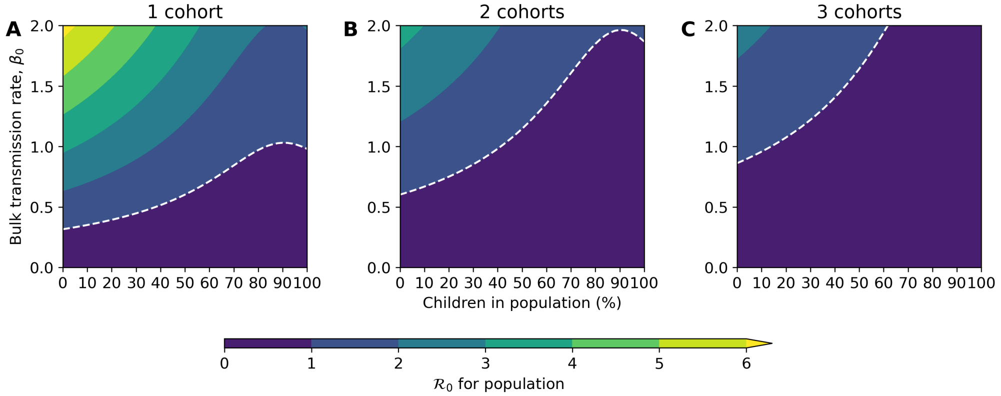

Fig S2. Interaction between bulk transmission rate and demography on short-term dynamics. Here $\beta_{ij} = \beta_0 \times f_{ij}$ with the choices $f_{11} = 0.1$, $f_{12} = 0.25$, $f_{21} = 0.15$, and $f_{22} = 0.5$. Increasing the proportion of children will necessarily increase the influence of child-specific parameters in the model. For example, here we have assumed child-child transmission is weak in relation to other interaction types so increasing the proportion of children paradoxically decreases $\mathcal{R}_0$.

Additional details on model sensitivity

Unless specified otherwise, model parameters are taken as defined in Table 1 in the main text.

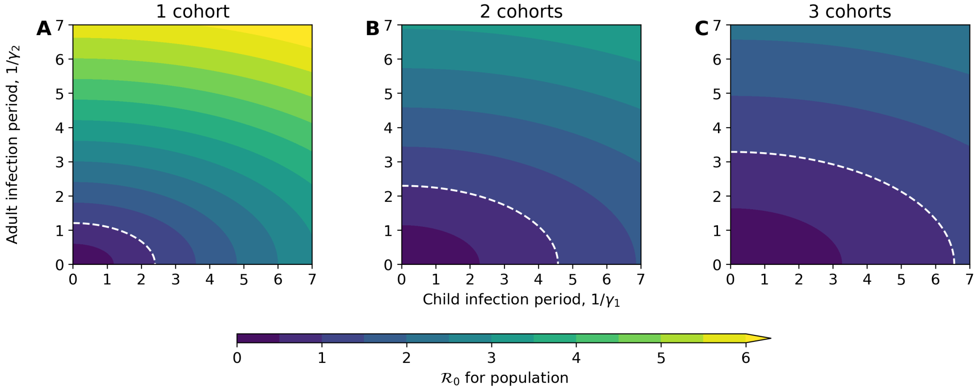

Fig S3. Influence of infectious period on short-term dynamics. White line highlights the threshold $\mathcal{R}_0 = 1$. Because the virtual population is taken as 22% children, the adult infectious period has a stronger influence on $\mathcal{R}_0$ compared to the child infectious period. The difference is neglible in the single cohort scenario but becomes pronounced as the number of cohorts increases.

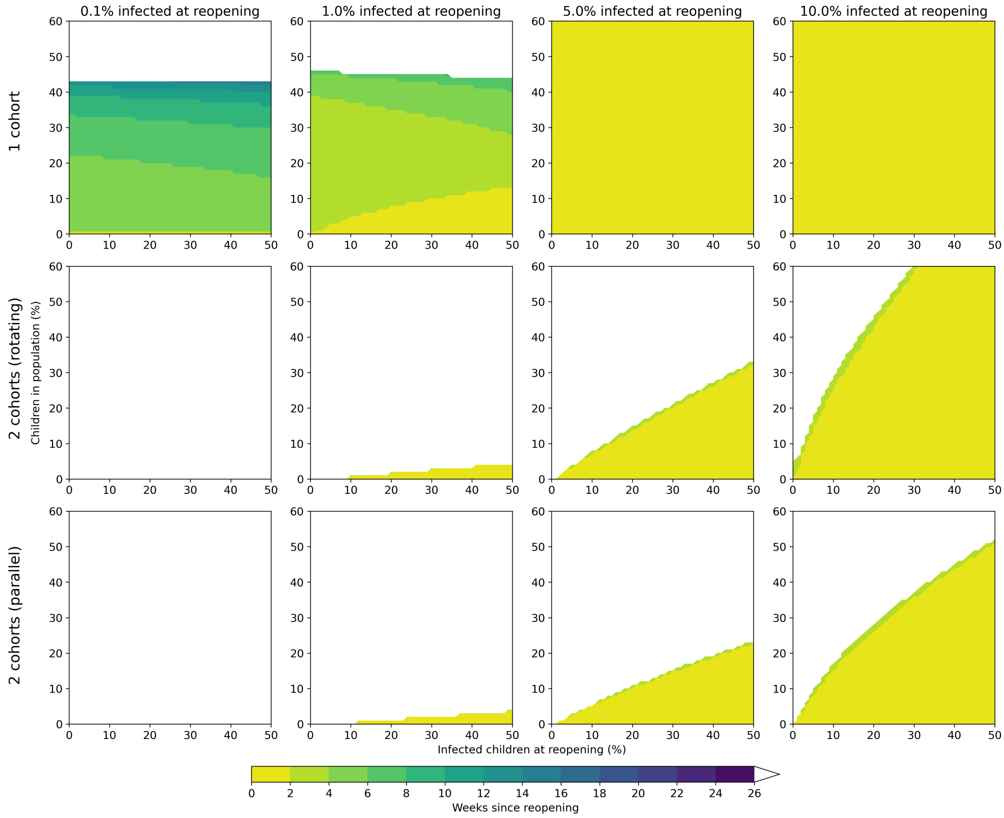

Fig S4. Interaction between initial proportion of children infected and demography on stopping time.

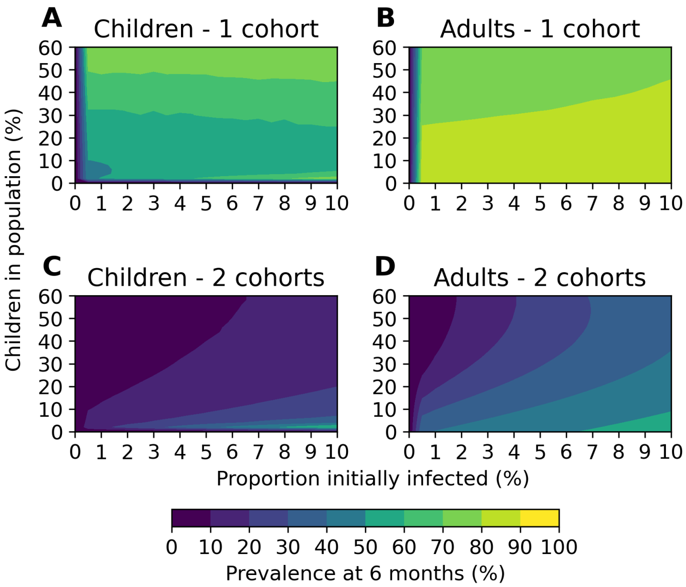

Fig S5. Interaction between initial proportion infected and demography on prevalence without testing.

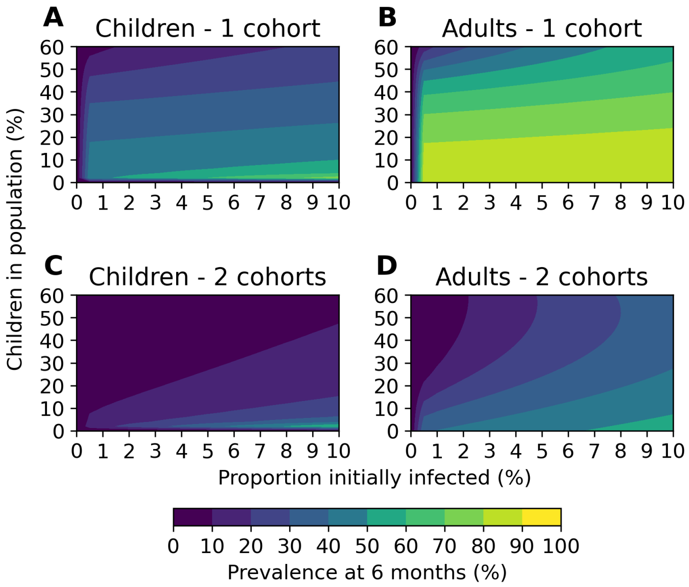

Fig S6. Interaction between initial proportion infected and demography on prevalence with testing.

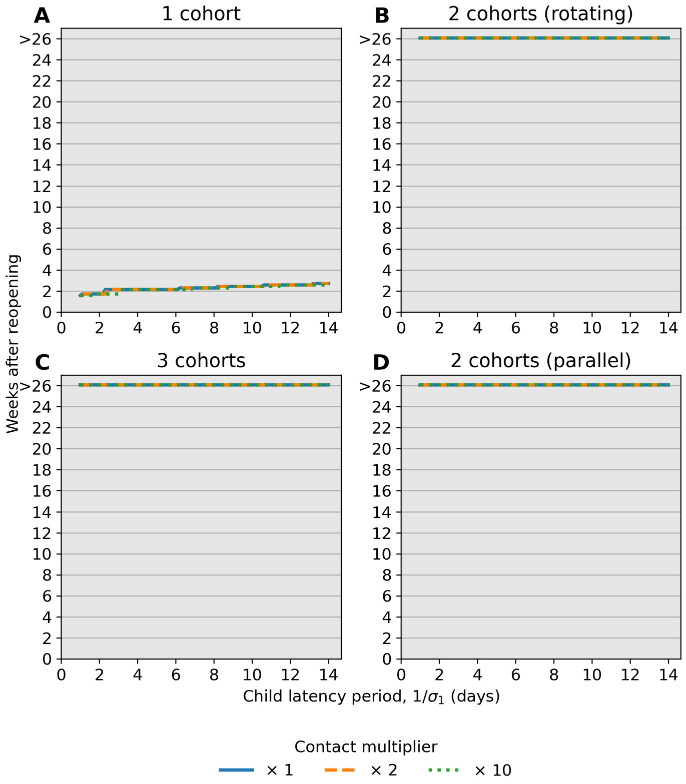

Fig S7. Interaction between child-child contact multiplier and child latency period on stopping time. Both the child latency period and child-child multiplier parameters have negligible effect on the stopping time, t_{thresh}. Here the initial proportion infected in the population is set to 2%, or 2000 active cases per 100,000.

References

1. Diekmann O, Heesterbeek JAP, Roberts MG. The construction of next-generation matrices for compartmental epidemic models. J R Soc Interface. 2010 Jun 6;7(47):873–85.

2. U.S. Department of Education, National Center for Education Statistics. American Community Survey Education Tabulation 2014-2018. https://data-nces.opendata.arcgis.com/datasets/acs-ed-2014-2018-children-enrolled-public-economic-characteristics-cdp03
